# Evidence and patterns of tuna spawning inside a large no-take Marine Protected Area

**DOI:** 10.1038/s41598-019-47161-0

**Published:** 2019-07-24

**Authors:** Christina M. Hernández, Jan Witting, Ciara Willis, Simon R. Thorrold, Joel K. Llopiz, Randi D. Rotjan

**Affiliations:** 1 Woods Hole Oceanographic Institution, Biology Department, Woods Hole, 02543 USA; 2 Sea Education Association, Woods Hole, 02543 USA; 3 Dalhousie University, Department of Biology, Halifax, B3H 4R2 Canada; 4 New England Aquarium, Anderson Cabot Center for Ocean Life, Boston, 02210 USA; 5 Boston University, Department of Biology, Boston, 02215 USA

**Keywords:** Ecology, Biooceanography, Biooceanography, Marine biology, Marine biology

## Abstract

The Phoenix Islands Protected Area (PIPA), one of the world’s largest marine protected areas, represents 11% of the exclusive economic zone of the Republic of Kiribati, which earns much of its GDP by selling tuna fishing licenses to foreign nations. We have determined that PIPA is a spawning area for skipjack (*Katsuwonus pelamis*), bigeye (*Thunnus obesus*), and yellowfin (*Thunnus albacares*) tunas. Our approach included sampling larvae on cruises in 2015–2017 and using a biological-physical model to estimate spawning locations for collected larvae. Temperature and chlorophyll conditions varied markedly due to observed ENSO states: El Niño (2015) and neutral (2016–2017). However, larval tuna distributions were similar amongst years. Generally, skipjack larvae were patchy and more abundant near PIPA’s northeast corner, while *Thunnus* larvae exhibited lower and more even abundances. Genetic barcoding confirmed the presence of bigeye (*Thunnus obesus*) and yellowfin (*Thunnus albacares*) tuna larvae. Model simulations indicated that most of the larvae collected inside PIPA in 2015 were spawned inside, while stronger currents in 2016 moved more larvae across PIPA’s boundaries. Larval distributions and relative spawning output simulations indicated that both focal taxa spawned inside PIPA in all 3 study years, demonstrating that PIPA is protecting viable tuna spawning habitat.

## Introduction

Tropical tunas are extremely valuable worldwide as a source of protein and income. Skipjack tuna alone provide approximately 50–60% of annual global tuna catches^1^. Pacific island nations earn a large proportion of their gross domestic product (GDP) from tuna, many by selling fishing licenses to the commercial fleets of foreign nations to operate in their exclusive economic zones (EEZ)^2^. One of these island nations is the Republic of Kiribati, which comprises 34 islands with a total land area of 810 km^2^ and an EEZ of 3.5 million km^2^, across 3 archipelagos that span 4.7°N to 11.4°S and 150.2°W to 187°W: the Line Islands, the Phoenix Islands, and the Gilbert Islands. For this low-lying ocean nation, tuna fishing by foreign commercial fleets is incredibly important to the economy: for example, from 2006 to 2015, fishing license revenue represented 39.5% of GDP on average, ranging from 19.2% to 93.5%^3,4^. Some of the variance in fishing license revenue can be attributed to the El Niño Southern Oscillation (ENSO) cycles. El Niño conditions tend to cause skipjack tuna, which dominate the catch in Kiribati waters, to move from the western Pacific warm pool into the central Pacific, and particularly into the Phoenix Islands region^5,6^. The year of highest contribution of fishing licenses to GDP, 2015, was an El Niño year, and Kiribati reported fishing license revenue of USD 148.8 million^3^.

Despite heavy reliance on tuna license revenues, approximately half of the Kiribati EEZ in the vicinity of the Phoenix Islands archipelago—and 11.3% of their total EEZ—is currently a no-take marine protected area (MPA) with UNESCO World Heritage Designation. The Phoenix Islands Protected Area (PIPA) is one of the largest marine protected areas in the world at 408,250 sq. km. Created in 2008 as a mixed-use MPA, and closed entirely to all commercial extractive activities in January 2015, PIPA comprises 8 atolls, 2 shallow submerged banks, at least 14 seamounts, and a large area of deep ocean^7,8^. This MPA was established to protect the many endangered and endemic species that live within its boundaries, as well as to protect the migratory birds, mammals, and sea turtles that pass through the area. Populations of previously exploited species, such as giant clam and coconut crab, have been recovering since the establishment of the MPA^8^.

In addition to biodiversity goals, the PIPA Management Plan lays out the hope that, if well-enforced, PIPA may protect tuna breeding stocks and potential spawning grounds. Although enforcing a no-take policy in an area the size of PIPA can be difficult, Automatic Identification System (AIS) data from ships indicates that virtually all fishing activity did indeed stop after January 1, 2015^7,9^. Furthermore, detected fishing days in (non-PIPA) Kiribati waters from AIS data actually increased from 2014 to 2015, indicating that fishing vessels moved out of PIPA but continued to fully subscribe fishing permit days for use in other parts of the Kiribati EEZ^6,7^. With effective enforcement inside PIPA, but heavy fishing pressure outside^9^, there may be value in protecting tuna spawning grounds, and potential for regional economic gain via “spillover effects” that may materialize once the closure has been in place long enough. Spillover effects occur when time- and/or area-closures result in increased biomass around MPA margins, which then moves outside the protected area where it can benefit regional fisheries, and these effects have been detected across a number of taxonomic groups and a range of MPA sizes^10–12^. For tunas in PIPA, it was assumed that fisheries protection of a large area where spawning occurs could have recruitment and biomass benefits in surrounding Kiribati waters and beyond, but tuna spawning activity within PIPA has not yet been confirmed.

Tropical tuna species that are likely to use the waters in PIPA for foraging and spawning include skipjack (*Katsuwonus pelamis*), yellowfin (*Thunnus albacares*), and bigeye (*Thunnus obesus*). Skipjack tuna are most abundant within 20° of the equator, but are found as far north as 40°N^13^. Yellowfin tuna are concentrated in equatorial waters and prefer temperatures above 25 °C^13^. Bigeye tuna, the largest and most valuable of the tropical tunas, have a range that extends from 40°S to 40°N^13^. Albacore tuna (*Thunnus alalunga*) may also pass through the region, but because of its subtropical to temperate habitat preferences, this species accounts for < 1% of the tuna catch in Kiribati^3^. Likewise, albacore larvae tend to be absent from equatorial waters and are more common at 20°N/S^14,15^.

Skipjack, yellowfin, and bigeye tuna are all fast-growing and early-maturing species, maturing at 1–3 years old^16–22^. In all three species, individuals are likely to spawn every 1–3 days over a period of weeks to months and, at the population level, spawning occurs throughout the year in the tropical Pacific^16–19,23,24^. For all three species, spawning and/or larval occurrence is generally observed at sea surface temperatures (SSTs) above 24 °C^19–22^.

Yellowfin and skipjack tuna larvae were found from 15°S to 23°N and across the full longitudinal range of sampling by NOAA expeditions in 1950–1952, from 110°W to 180°W^23,24^. The only other broad-scale sampling effort for larval tunas in the Pacific Ocean was carried out by the Japanese from 1956–1981. In these collections, the larvae of skipjack, yellowfin, and bigeye tunas were broadly distributed in the western and central Pacific from approximately 20°S to 30°N^14,15^. There have been a number of other larval studies of tropical Pacific tunas, but they focused on distributions in the vicinity of islands and atolls and include limited sampling effort in deep pelagic zones^25–27^. Larvae of many tuna species are known to concentrate in the upper 50 m of the water column^14,23,28,29^.

**Figure 1:**
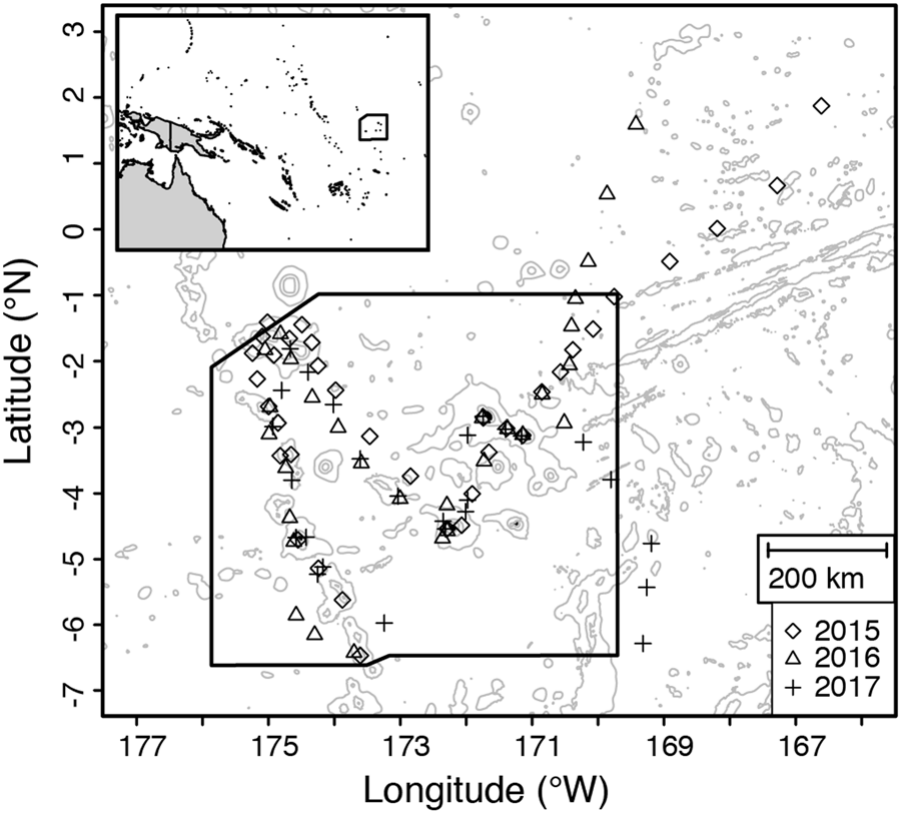
Map of sampling locations. For the cruises in 2015, 2016, and 2017, locations of all samples included in these analyses are shown. Bathymetric contours are shown at 1000, 3000, and 5000 m depth (accessed through GEBCO). The inset map shows the location in reference to Australia and the island of New Guinea. In both the main plot and the inset, the solid black line shows the boundaries of PIPA.

In this study, we combined empirical data on larval tuna abundance and growth, collected on annual cruises to PIPA in July/August in 2015–2017 (Fig. 1), with individual-based model simulations to estimate spawning locations and relative spawning output. We estimated, for the first time, abundance of tuna larvae in PIPA waters and determined which species of tuna are spawning within and around PIPA, confirmed with genetic barcoding for species within the genus *Thunnus*. Our data set includes the 2015 El Niño event that, along with global elevated temperatures, led to widespread coral bleaching across the tropical Pacific^30^. In the summers of 2016 and 2017, we determined conditions to be ENSO neutral. Because *Thunnus* spp. tunas show preferences for spawning in highly oligotrophic waters^31^, we expected to find higher larval abundances under the low chlorophyll El Niño conditions in 2015 than in the more productive conditions in 2016 and 2017.

## Results

### Oceanographic conditions

**Figure 2:**
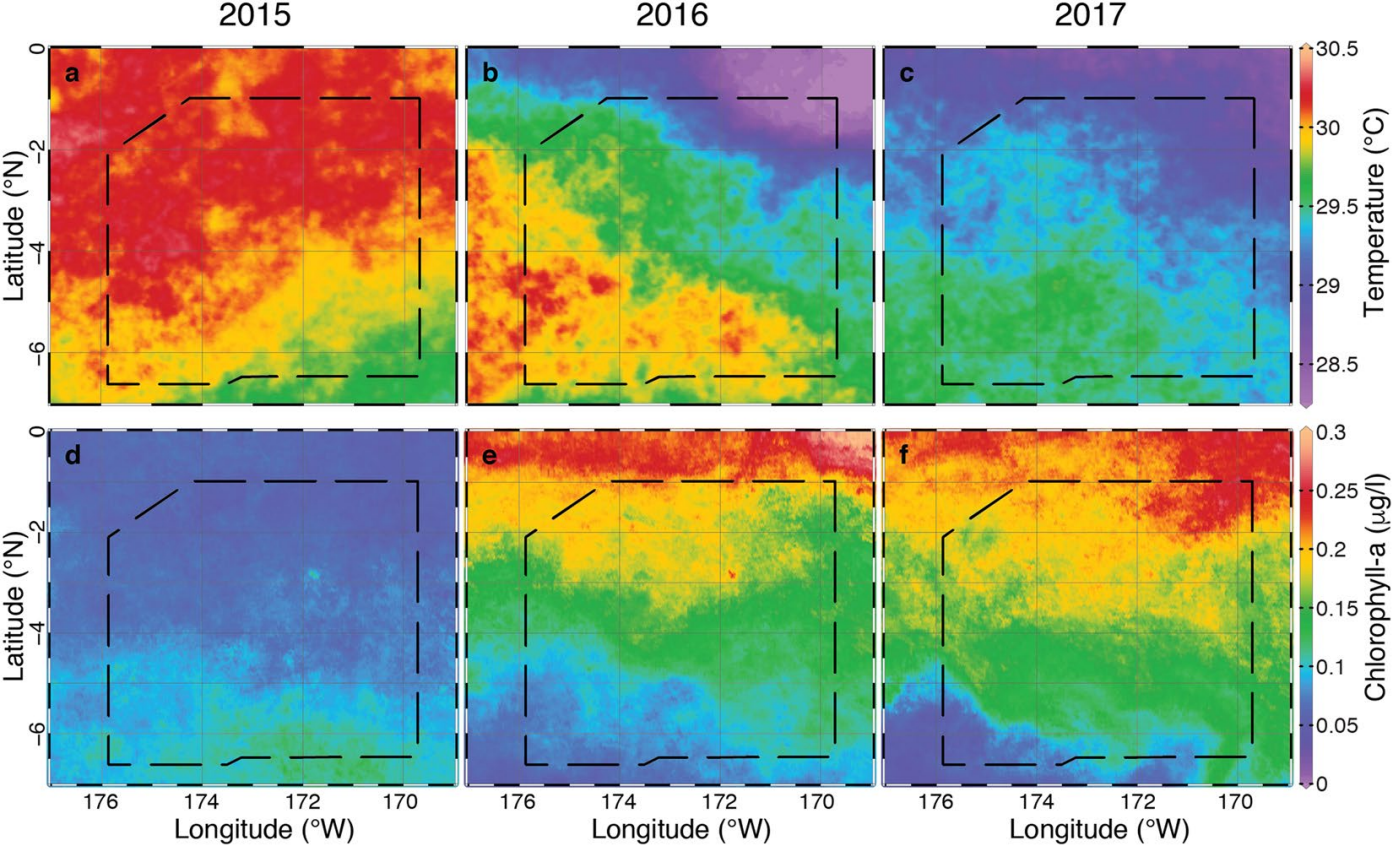
Environmental conditions across the three years of sampling. Panels show satellite-derived sea surface temperature (**a–c**) and chlorophyll (**d–f**) for 2015 (**a,d**), 2016 (**b,e**), and 2017 (**c,f**). The dashed lines show the boundaries of PIPA. Sea surface temperature data (°C) comes from the MURSST dataset, and chlorophyll data (μg/l) comes from the Visible and Infrared Imager/Radiometer Suite (VIIRS) satellite (both datasets through NASA).

The surface mixed layer in PIPA extended to approximately 100 m with temperatures above 25 °C, below which there was a gradual thermocline to temperatures below 12 °C at 300 m (Fig. S3**)**. In 2015 (El Niño year), the surface waters in PIPA were warmer than in 2016 and 2017 at the northern boundary by about 1–2 °C, while at the southern boundary, temperatures were quite similar in 2015 and 2016 (Fig. 2a,b). In 2017, surface temperatures were fairly uniform across the protected area, and slightly cooler than in 2016 (Fig. 2c). From 2015 to 2016, there was a marked difference in sea surface chlorophyll concentrations in PIPA, both in terms of the typical value and a reversal in the latitudinal gradient. Surface chlorophyll plots for 2015–2017 clearly demonstrate the broad extent of low relative productivity in 2015 (Fig. 2d–f). In 2015, chlorophyll values were below 0.15 μg/l throughout all of PIPA, and the higher values (between 0.1 and 0.15 μg/l) were found only around Kanton atoll and in the southern half of PIPA (Fig. 2d). In 2016, chlorophyll values were above 0.1 μg/l throughout nearly all of PIPA, with the exception of the southwest corner; the highest values of over 0.2 μg/l were near the northern boundary and values decreased with distance from the equator (Fig. 2e). The pattern of surface chlorophyll in 2017 resembled that in 2016 (Fig. 2f).

**Figure 3:**
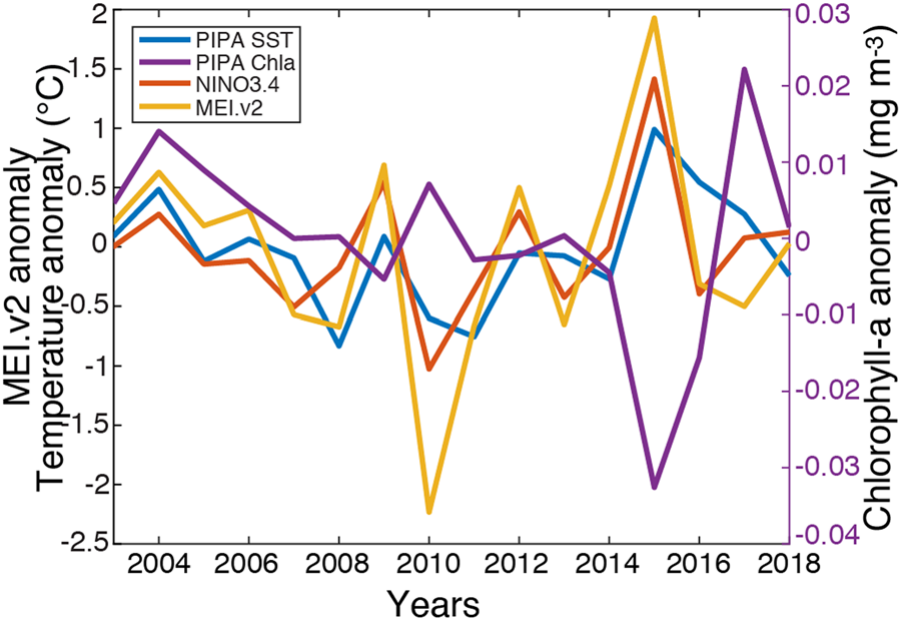
Environmental conditions in PIPA for 2003–2018. July SST anomaly in PIPA (°C) was calculated from daily MUR SST data. July chlorophyll-a concentration anomaly in PIPA (mg m^−3^) was calculated from daily MODIS Level 3 data. Also plotted are the July Nino3.4 anomaly and the July Multivariate ENSO Index (MEI) anomaly.

Compared with July SST from 2003–2018, the July SST in PIPA in 2015 was the hottest, with an anomaly of nearly 1 °C from the 2003–2018 mean. The July SST in 2016 and 2017 were also slightly warmer than the 2003–2018 average climatology, with anomalies of 0.55 and 0.28 °C, respectively (Fig. 3). The July SST from PIPA generally tracks the Niño3.4 index and the Multivariate ENSO index (MEI) (r = 0.68 and 66, respectively), although those indices both indicated cool conditions in July of 2016 and 2017, while the PIPA temperatures showed weak positive anomalies. In years with strong signals in the MEI (2010 and 2015), the chlorophyll-a concentration in PIPA moved out of phase with SST. Compared with the rest of the 15-year time series, the July chlorophyll-a concentration in PIPA had its lowest value in 2015 and its highest value in 2017 (Fig. 3).

### Larval tuna abundance and distribution

**Figure 4:**
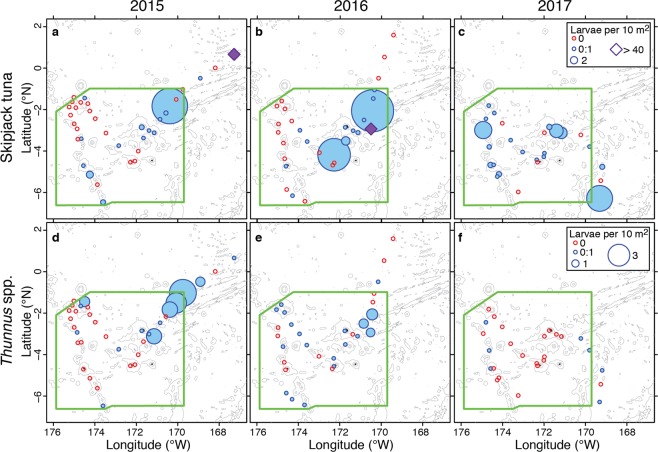
Larval tuna distribution maps. Subpanels depict abundance for *Katsuwonus pelamis* (panels a–c) and *Thunnus spp*. (panels d–f). Panels a and d show the larval distribution in 2015, panels b and e for 2016, and panels c and f for 2017. Red circles indicate locations that plankton sampling occurred but no larvae of that taxon were collected. The size of blue circles is scaled to the abundance of larvae at that station, and purple diamonds in panels a and b correspond to an anomalously high station abundance (42.5 and 40 larvae per 10 sq. m in a and b, respectively). The boundaries of PIPA are shown in green. Bathymetric contours at 1000, 3000, and 5000 km depth are shown in light grey (accessed through GEBCO).

Skipjack and *Thunnus* spp. larvae were caught in all 3 years, for a total of 64 non-zero stations (Table S1) and 42 stations with zero catch. In 2015, 163 skipjack larvae were caught in the study area; 59 of those inside PIPA, and a large catch of 101 skipjack larvae occurred outside of the northeastern corner of PIPA. The abundance of skipjack larvae at non-zero stations in 2015 ranged from 0.004 to 42.5 larvae per 10 m^2^ (Fig. 4a). In 2016, 291 skipjack larvae were caught in the study area, all inside the PIPA boundaries, and a large catch of 184 skipjack larvae occurred in the northeast quadrant of PIPA. The abundance of skipjack larvae at non-zero stations in 2016 ranged from 0.22 to 40.75 larvae per 10 m^2^ (Fig. 4b). In 2017, 82 skipjack larvae were caught; 72 of those were caught inside of PIPA. There was no single station with a markedly high catch, but in 2017 no samples were collected in the farther northeast region where high catches were observed in 2015 and 2016 (Fig. 4). The abundance of skipjack larvae at non-zero stations in 2017 ranged from 0.004 to 7.12 larvae per 10 m^2^ (Fig. 4c).

In 2015, 39 *Thunnus spp*. larvae were collected in the study area and 34 of these were collected inside of PIPA. The abundance of *Thunnus spp*. larvae at non-zero stations in 2015 ranged from 0.009 to 3.7 larvae per 10 m^2^ (Fig. 4d). In 2016, 35 *Thunnus spp*. larvae were collected in the study area and 34 of these were caught inside PIPA; the abundances at non-zero stations ranged from 0.25 to 1.5 larvae per 10 m^2^ (Fig. 4e). In 2017, 8 *Thunnus spp*. larvae were caught in the study area, and 6 of these were caught inside PIPA; the abundances at non-zero stations ranged from 0.005 to 0.89 larvae per 10 m^2^ (Fig. 4f).

Overall, the abundance of skipjack tuna larvae was higher but more variable than the abundance of *Thunnus spp*. larvae (Fig. 4). Abundances of skipjack larvae were higher in 2016 than 2015, while for *Thunnus* spp., larval abundances in 2015 and 2016 were similar. Abundances of both taxa were lower in 2017 than in the previous two years.

### DNA barcoding

Due to morphological characters being unreliable for distinguishing yellowfin (*Thunnus albacares*) and bigeye (*Thunnus obesus*) tuna larvae^32^, DNA barcoding was used on a subsample of larvae for species identification. These analyses positively identified both bigeye and yellowfin larvae within PIPA. In 2015, six were identified as bigeye tuna and 18 as yellowfin tuna. In 2016, nine were bigeye tuna and no other *Thunnus* species were identified. In 2017, one matched with bigeye tuna and two with yellowfin tuna. All seven of the barcoded *Thunnus* eggs were from bigeye tuna (five from 2015 and two from 2016). Upon examination of a subset of the genetically identified larvae, we confirmed that morphological features were not useful for distinguishing bigeye from yellowfin larvae (details in SI).

### Spawning sites and relative output

Particle backtracking was performed using a coupled biological-physical dispersal model to estimate the spawning locations and relative output that contributed to our observed larval collections. We calculated relative spawning output by scaling the probability distribution of spawning locations for each larva by its age, using a temperature-based mortality rate, and then combining these scaled distributions by taxon and year.

**Figure 5:**
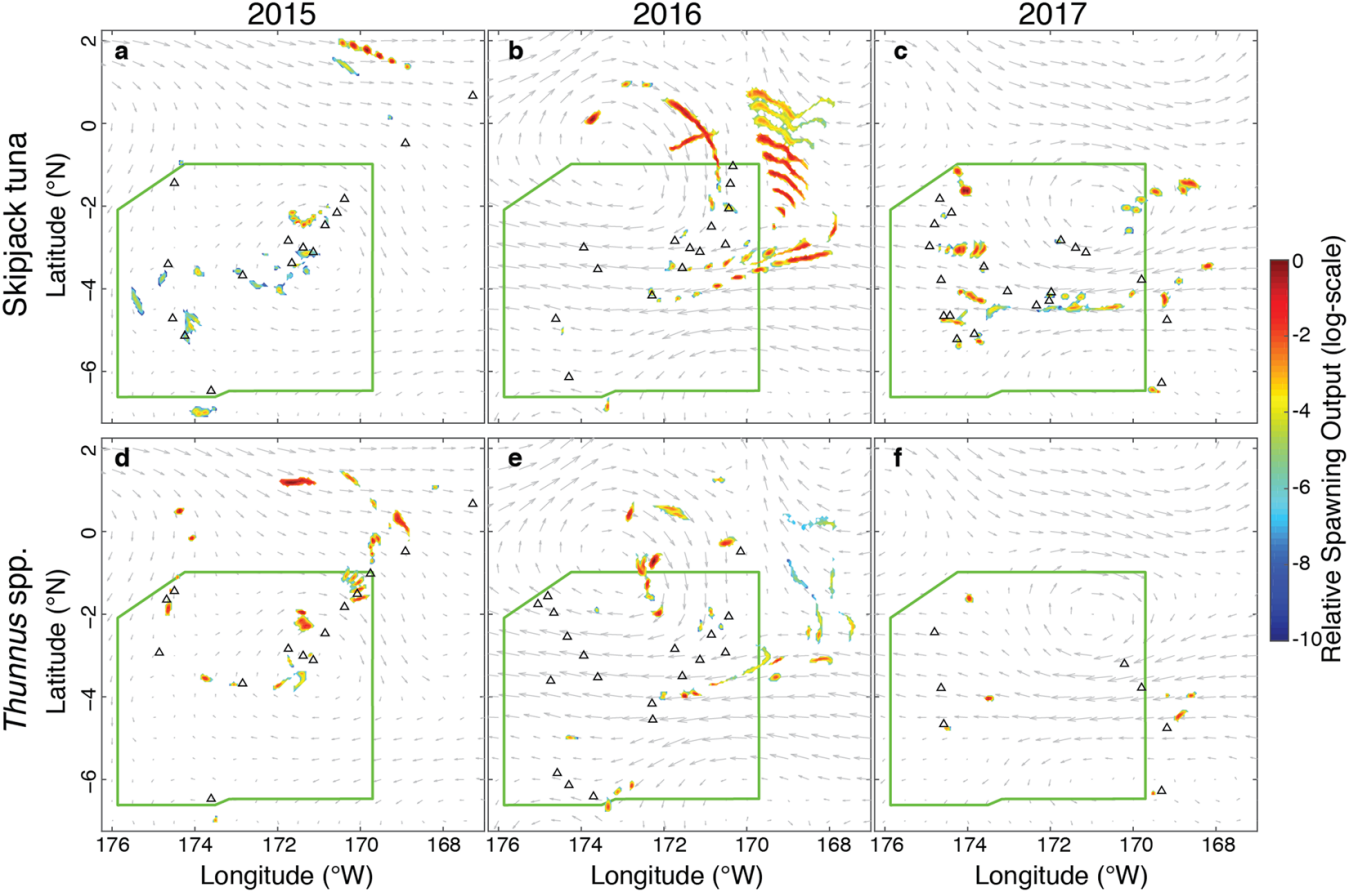
Relative spawning output map. Relative spawning output, from backtracking simulations, is shown for *Katsuwonus pelamis* (panels a–c) and *Thunnus spp*. (panels d–f). Panels a and d show the relative spawning output in 2015, panels b and e for 2016, and panels c and f for 2017. The log-scale colorbar corresponds to filled color contours of relative spawning output, which was calculated on 0.05° by 0.05° latitude/longitude grid and normalized such that the largest value in each panel is 1. Black triangles indicate the release locations for backtracking simulations, which are the same as the collection locations in Fig. 3. The boundaries of PIPA are shown in green. Current vectors represent the mean HyCOM velocities in the upper 25 m of the water column for the sampling period of each year.

In 2015, velocities of modeled currents were generally low with a weak anti-cyclonic retention feature present inside PIPA, centered around Kanton atoll (Fig. 5a,d). The larvae collected in 2015 inside PIPA generally originated from spawning that occurred inside of PIPA, while those collected outside the boundaries generally originated outside. The exceptions to this were the stations nearest to the protected area boundaries, including the southernmost station inside PIPA, the two stations near the northwest corner, and the station at the northeast corner. In 2016, modeled currents were stronger and there was more movement of eggs and larvae across the PIPA boundaries (Fig. 5b,e). In the mean state of the currents over the sampling period, an anti-cyclonic circulation feature was present to the north of PIPA, with westward currents cutting through the protected area near the middle of its latitudinal range. As a result, inferred spawning activity was more spread out along the prevailing currents. In 2017, the modeled currents were intermediate in strength; there was an anti-cyclonic retentive feature in the northeast quadrant and westward currents at the middle latitude of the protected area (Fig. 5c,f). The larvae collected in the western half of PIPA were likely from spawning inside the boundaries, while those collected in the eastern half likely originated near the eastern boundary. The larvae collected outside the eastern boundary of PIPA were likely from spawning outside of PIPA.

## Discussion

By combining three years of empirical data on larval tuna in PIPA with a biophysical modeling approach to backtrack larvae to infer spawning locations, we have conclusively demonstrated that spawning occurs inside PIPA for skipjack, bigeye, and yellowfin tunas. Moreover, this finding held during both El Niño and neutral conditions. The overall pattern of larval distribution and abundance was similar across years, and backtracking simulations suggested that spawning locations near PIPA were also similar across years. Larval studies are valuable because they reveal both adult presence and spawning activity without removing adults or interrupting spawning behavior. Therefore, this study can help us understand the role that PIPA plays in tuna conservation, while adhering to the no-take policy of the protected area management plan.

The larval tuna abundances that we recorded in PIPA, at non-zero catch stations, range from 0.004 to 42.5 larvae per 10 m^2^, or approximately 0.008 to 85 larvae per 1000 m^3^ (assuming that larval habitat is the upper 50 m). Past work on larval tuna distributions in the Pacific is limited, but the abundances presented here are within the range of previous studies. For example, the 1950–1952 surveys by NOAA in the pelagic tropical Pacific found 1–16 yellowfin tuna larvae per 1000 m^3^ and 0.5–25 skipjack tuna larvae per 1000 m^3^ ^23,24^. The other example of broad-scale larval surveys in the tropical Pacific, undertaken by the Japanese from 1956–1981, observed larval yellowfin at densities of less than 1 per 1000 m^3^ and skipjack tuna larvae at densities of 0.1–5 per 1000 m^3^ ^14,15^. Sampling around Johnston atoll found *Thunnus* spp. larvae at 0.1–0.3 per 1000 m^3^, and skipjack larvae at 0.3–1.7 per 1000 m^3^ ^26^. Sampling around the Hawaiian archipelago yielded 1–10 larvae per 10 m^2^ for *Thunnus* spp. and 0.1–1 skipjack larvae per 10 m^2^ ^25^. One study, in the waters around French Polynesia, found very high concentrations of tuna larvae; they observed a maximum station abundance of 446 *Thunnus* spp. larvae per 1000 m^3^, and a median of approximately 20 per 1000 m^3^, and skipjack tuna larvae had a median of about 8 per 1000 m^3^ ^27^. Our results fit well into the established literature on larval studies in the tropical Pacific, suggesting that spawning in PIPA likely contributes to recruitment in central Pacific tuna stocks.

Although July of 2015 had the warmest and lowest chlorophyll-a conditions that were seen in the month of July between 2003 and 2018 (Fig. 3), the overall pattern of abundance of tuna larvae did not change substantially between 2015 and the other sampling years (Fig. 4). The largest inter-annual difference was the much lower range of abundances in 2017. However, this coincides with lower effort in 2017, particularly in the northeast quadrant of PIPA. If we compare only the locations where sampling occurred in all three years, then abundances reported for 2017 are quite similar to the other two years.

Although we observed consistency in the overall patterns of larval abundance across years, there were differences in the details of those patterns. We expected to find higher larval abundances under low chlorophyll El Niño conditions because of the preference of *Thunnus* spp. tunas for spawning in highly oligotrophic waters^31^. The observed abundance of *Thunnus* spp. larvae was indeed higher—but patchier—in 2015 than in 2016. In 2016, there were more positive stations but abundance was generally lower at each station (Fig. 4d,e). In 2015, barcoding results suggest the presence of both yellowfin and bigeye tunas, but in 2016 only bigeye tuna were identified (Table S1). Unfortunately, species identification with DNA barcoding was not possible for 74% of the *Thunnus* spp. larvae collected in 2016, so the possibility remains that yellowfin occurred in 2016 as well. (The quantity of pure ethanol brought in 2016 was insufficient for preserving the high amounts of plankton that occurred. As such, DNA was often degraded in these samples, or denatured ethanol—a poor DNA preservative—was used instead.) Although surface temperatures were cooler in 2016, the entire area of PIPA had surface temperatures above 28 °C (Fig. 2b), well within the preference range of yellowfin tuna^13^. If there were truly no yellowfin larvae in our 2016 collections, it could indicate that bigeye tuna distribution and spawning activity is less responsive to changes in the surface waters than these aspects of yellowfin tuna biology. This, in turn, could be explained by observations that bigeye tuna often occupy colder waters and greater depths^13,33^, and forage on deeper-dwelling organisms than do yellowfin tuna^34^.

Another important component of interannual variability is the influence of currents on the pattern of relative spawning output. The backtracked reproductive output maps show little movement of eggs and larvae across the boundaries of PIPA during El Niño conditions (i.e., 2015), and greater movement during neutral conditions. For example, the areas of highest relative spawning output for skipjack tuna were outside PIPA to the north in both 2015 and 2016 (Fig. 5a,b), but the observed large catch locations were outside PIPA in 2015 and inside in 2016. This is due to weaker currents accompanied by a northerly position of the mesoscale circulation feature in 2015 compared to stronger currents and a more southerly mesoscale feature in 2016. In both 2016 and 2017, there is a westward current flowing near the middle latitude of PIPA. This is in line with observations of the westward-flowing southern branch of the South Equatorial Current, which is centered around 3°S and weakens during El Niño events^35^.

We used larval catch, ages, and estimated mortality rates to take a novel approach to larval backtracking. In addition to showing that many of the tuna larvae originated from spawning activity in PIPA, we also shed light on spatial variability in spawning output. However, it is important to note that these results are based only on the observed collections and, as such, the backtracking results should be thought of as presence-only—spawning may have occurred in more of the domain, with larvae transported to locations where we did not sample. Furthermore, the results of individual-based modeling are quite sensitive to the choice of hydrodynamic model. We used HYCOM, a data-assimilative hindcasting model that generally replicates large-scale patterns in the ocean, and we find that it matches CTD data from PIPA reasonably well (Fig. S2). However, further work on hydrodynamic models for the tropical Pacific would improve the reliability of larval dispersal, connectivity, and backtracking simulations that are often used in MPA design and assessments.

Studies of larval tuna abundance and its interannual variability can contribute substantially to our understanding of tuna spawning, stock productivity, and the role of large MPAs like PIPA in tuna conservation. While our results show that tuna are spawning inside of PIPA, the lack of other larval tuna studies in the tropical Pacific limits our ability to contextualize the role of PIPA in overall stock productivity. The only broad-scale larval tuna studies in the tropical Pacific ended between 40^15^ and 70 years ago^23,24^. Still, given the remoteness of PIPA, as well as its size, this work represents a significant contribution to the scientific understanding of tuna spawning in open waters of the Pacific and to the use of protected areas by highly migratory species.

Recently, several studies have focused on the role of protected areas, including large-scale MPAs, in the conservation and management of highly migratory species. Around the long-standing Galápagos marine reserve, there is evidence of “spillover effects” in the yellowfin tuna purse seine fishery, indicating that the protected area is increasing recruitment locally—whether by protecting a fairly resident population or by attracting very large temporary aggregations of yellowfin^36^. In a different management context, temporary closures of the striped marlin long-line fishery in Baja California coincided with rapid increases in fish abundance, again with some uncertainty about whether the increase is driven by high recruitment of a local population or import of individuals from outside the closed area^37^. In a counter example, it was deemed unlikely that a proposed MPA at Ascension Island (tropical Atlantic) would lead to population benefits for the yellowfin tuna population; although individuals spent several months at a time foraging around the island, they were not found to be reproductively active^38^. In an example from a highly migratory species with a very different life cycle, tagging studies on green turtles in the Chagos Archipelago MPA indicate that turtles spend a very small proportion of time inside the MPA, using it for nesting but not for foraging^39,40^. In all of these examples, the value of the protected area comes from the combination of adult presence and reproductive activity.

We have demonstrated that there are reproductively active individuals within PIPA, but a critical piece of missing information that would allow for a better assessment of the protection afforded by PIPA would be the residence times of adult tuna. Otolith chemistry, which is related to natal origin using geographically-based water properties, suggests that both yellowfin and bigeye tuna populations in the tropical Pacific are structured on large regional scales^41^. Likewise, tagging data suggest stock structure and regional retention on large spatial scales for bigeye tuna^42^ and that yellowfin tuna will remain in an area similar in size to PIPA for periods of weeks to months^38,43^. A global synthesis of dart tag data indicated that all three tropical tuna species are capable of both rapid large-scale movements and regional fidelity or periodic returns to feeding and spawning locations^44^. This evidence suggests that, if foraging conditions in PIPA are attractive, adult tuna could spend enough time there to benefit from the protection in order to spawn numerous times before becoming vulnerable to fishing again. A tagging study specifically focused on adult tuna movements in the vicinity of PIPA would further elucidate the scale of PIPA’s impact on tuna population productivity.

In addition to short-term contributions to sustainable management, PIPA may be important for the conservation of tuna populations affected by climate change. Several papers have predicted that climate change will have neutral effects or benefits for tuna fisheries in island nations in the central Pacific Ocean^2,45^. Climate projections indicate that tuna abundances in PIPA, and in Kiribati in general, will increase because warming in the western Pacific warm pool will drive the population distribution of skipjack tuna towards the east^46,47^. Likewise for bigeye tuna, climate change projection models predict that adult abundances will increase in the eastern Pacific and will decrease in the western and central Pacific^48^. Part of the reasoning is that climate projections indicate that spawning habitat for skipjack tunas in the western Pacific will decrease substantially^48,49^. Thus, under certain climate scenarios, PIPA may have a disproportionate opportunity to bolster reproductive stocks if surface water temperatures cause adults to spend more time within the MPA boundaries.

The Phoenix Islands Protected Area is unique as a large, continuous MPA that encloses almost an entire archipelago and encompasses both shallow reef environments and deep pelagic waters. Larval studies, including the analysis of distribution, abundance, growth, and backtracking simulations, contribute to overall understanding of the species and/or the stock because events in early life can have an outsized impact on stock recruitment and productivity. However, linking a study of the distribution of tuna larvae in PIPA with the population dynamics of adult skipjack, yellowfin, and bigeye tunas in the central Tropical Pacific is difficult, and should be approached cautiously. The great power of the standard ichthyoplankton sampling scheme presented here is that it provides the baseline for a growing time series that, as annual sampling continues through at least 2022, will enable monitoring of tuna spawning inside PIPA. This time series may reveal interannual changes in the spawning output or shifts in its spatial distribution, providing insight into how tuna spawning in and around PIPA is likely to change under climate change conditions. Furthermore, the protection of large areas like PIPA, where the only major anthropogenic effects are from global-scale climate change, potentially enables us to disentangle climate-related impacts from the other multiple stressors that most ecosystems face. In addition to being a reservoir for biodiversity and biomass, mega-MPAs like PIPA are large-scale living laboratories that can help scientists better understand ocean ecosystems.

## Methods

### Field sampling

Sampling took place aboard the *SSV Robert C. Seamans* following similar cruise tracks and dates in 2015 (July 18 to August 7), 2016 (July 15 to August 6), and 2017 (July 17 to August 4) (Fig. 1). A few stations were sampled on transits from Honolulu, HI to PIPA (2015 and 2016) and between Pago Pago, American Samoa and PIPA (2017). Of the stations outside PIPA, only those reasonably close to the PIPA boundaries are included here, i.e. all stations between 7°S and 2°N (Table S1). Sampling beyond this latitudinal range is excluded from this study, including 7 stations in 2015 that yielded a total of 1 skipjack tuna larva; 8 stations in 2016 that yielded a total of 9 skipjack larvae (from 4 stations) and 2 *Thunnus* spp. larvae (from 1 station); and 9 stations in 2017 that together yielded 1 *Thunnus* sp. larva.

Station work occurred at approximately local noon and midnight each day. At each station, the hydrographic rosette, equipped with conductivity, temperature, and depth (CTD) sensors, was deployed to 600 m depth or 10 m off the bottom. To sample ichthyoplankton, up to 3 different types of plankton net tows were performed, with all 3 used at the majority of stations (Table S1). Net tows were performed for approximately 30 minutes at a speed of 2 knots. First, a 1-m^2^ rectangular opening Tucker trawl with a single net of 333 μm mesh was towed obliquely to the typical depth of the thermocline (approximately 100 m). Second, the same Tucker trawl was re-deployed for a shallower, double-oblique tow to approximately 50 m, intended to sample larval tuna habitat more intensively. Third, a 0.5 × 1 m rectangular neuston net was deployed off the side, sampling the upper 0.25 m.

The Tucker Trawl nets were equipped with an internally recording depth sensor and a General Oceanics flow meter, giving tow depth and volume filtered. Neuston tow flow was estimated using vessel speed, net area, and tow duration. Samples were fixed in 95% ethanol.

In addition to our *in-situ* environmental data, we mapped the average July surface temperature from the Multi-scale Ultra-high Resolution (MUR) SST data set^50^ and sea surface chlorophyll data from the Visible and Infrared Imager/Radiometer Suite (VIIRS) satellite^51^. To compare environmental conditions within PIPA beyond our sampling years, we used the MUR SST dataset^50^ and the MODIS chlorophyll-a dataset^52^. Using daily data for July in 2003–2018, we calculated the mean values in PIPA (latitude 6.6°S to 0.98°S and longitude 175.8°W to 169.7°W). These two time series are compared with the Niño3.4 index^53^ and the Multivariate ENSO index^54^. All four time series are represented as anomalies by subtracting the 2003–2018 average value.

### Lab processing

Fish larvae and fish eggs were separated from the bulk plankton samples under a light microscope. Using morphological characters, tuna larvae were identified as skipjack tuna (*Katsuwonus pelamis*) or *Thunnus* sp.^32,55^. Fish larvae and eggs were stored in 95% ethanol.

Abundance of larval tuna for each net tow (*a*_*net*_, n larvae per m^2^) was calculated by multiplying the density (n larvae per m^3^) in each net tow by the vertical distance it sampled. The standardization by vertical distance is required to combine multiple net observations, which each sampled different depths, into a single value of abundance per station. Then, the station abundance was calculated by taking the mean *a*_*net*_ of the two Tucker trawl tows and adding the neuston *a*_*net*_.

The two expected species of *Thunnus* larvae in our samples were bigeye tuna (*Thunnus obesus*) and yellowfin tuna (*T. albacares*)^14^. These two species have limited morphological characters that can differentiate them, and larvae under 5 mm are not well described. As such, species identification was confirmed genetically on a subset of *Thunnus* larvae by the Canadian Center for DNA Barcoding. Additionally, a subset of eggs that were visually identified as possible scombrid eggs were barcoded to empirically confirm spawning within PIPA. Barcoding sequences were obtained for 36 *Thunnus* larvae and 7 *Thunnus* eggs. Most of these could be classified using the Barcode of Life Database’s (www.bold.org) ‘Species DB’ search function, but manual inspection and classification of sequences was required for 11 of the 36 barcoded larvae (further details in SI).

Photographs were taken of each larva using a Leica M205C stereomicroscope with a Canon EOS 60D camera attached. Fish standard lengths were measured using ImageJ with the ObjectJ plug-in (https://imagej.nih.gov; https://sils.fnwi.uva.nl/bcb/objectj). Some fish larvae were damaged and could not be measured; however 191, 256, and 80 larvae (93, 75, and 85 percent of collection) were successfully measured from 2015, 2016, and 2017, respectively. Length of damaged larvae was estimated using the mean length for that taxon in that sample. If the sample contained fewer than 3 measurable larvae of that taxon, then the mean length from all nets at the collection station was used, weighted by the number of larvae in each net (5 occasions); if this still contained fewer than 3 larvae of that taxon, then the two nearest stations were combined with the collection station to calculate the weighted mean length (17 occasions).

Length-at-age relationships were constructed using otolith-derived ages from skipjack and *Thunnus* spp. larvae from 2015 and 2016 collections. Otoliths were extracted and read from 118 tuna larvae total: 36 and 30 skipjack and 25 and 27 *Thunnus* spp. from 2015 and 2016, respectively. After quality control, otolith reads were retained for 32 and 29 skipjack larvae and 22 and 26 *Thunnus* spp. larvae from 2015 and 2016, respectively. Aged larvae ranged from 2.24 to 9.04 mm in length and from 1 to 12 daily otolith increments. More details on otolith methods are in the SI.

A least squares linear regression was fitted to the daily increment and length data for each year-taxon pair, as well as by taxon pooled across years (Fig. S1). An ANOVA did not find a significant effect of year on larval length for skipjack tuna larvae (p = 0.22), and there was a marginally significant effect for *Thunnus* spp. larvae (p = 0.06). We found that average growth rates over the first two weeks of life are 0.45 and 0.37 mm per day for skipjack tuna larvae and *Thunnus* spp. larvae, respectively (Fig. S1). The size-at-age relationships derived from otolith analyses were used to assign the number of daily growth increments for larvae that were not aged.

To inform backtracking, we needed to estimate ages in days post spawning. It takes 18–30 hours for tuna eggs to hatch at the observed temperatures^22,56^. Yellowfin and skipjack tuna larvae begin to lay down increments within 12–24 hours of hatching^57,58^. Therefore, we added 2 days to convert the estimates of increment number into age of each larva in days post-spawning.

### Relative spawning output estimation

For particle backtracking, we used the Connectivity Modeling System (CMS)^59^ with velocities from the Hybrid Coordinate Ocean Model (HYCOM) Global hindcast experiments 91.1 and 91.2. The HYCOM data server provides daily currents at a horizontal grid resolution of 1/12°. For each station at which tuna larvae were caught, 1000 virtual particles were released at noon on the calendar day of sampling at the mean geographical position of all net tows for that station (Table S1). Virtual particles were released at 25 m depth, and passively drifted at fixed depths: 10 m depth for *Thunnus spp*. larvae and 25 m depth for skipjack larvae. These depths were selected because they represent the mid-point of observed vertical distributions for these taxa^28,29,60^. The particles were tracked backwards in time for 15 days from collection date, with a model time step of 20 minutes. The model includes stochastic kicks to simulate sub-grid scale diffusivity, and releasing 1000 particles ensures that the output will broadly sample that probability space.

Using the simulation output, we built a dataset corresponding to all collected tuna larvae. For each larva, age in days post spawning was estimated using the pooled size-at-age relationships. In order to account for variance in size-at-age, we drew the estimated ages from a normal distribution with mean equal to the regression line for age-at-size and variance equal to the standard deviation of all of the residuals of age-at-size. We use this age for each larva to extract the positions of the 1000 virtual particles (from the corresponding simulation release point) on the estimated day of spawning. We calculated the 2-D frequency distributions of the 1000 particle positions at the estimated time of spawning, on a 0.05° latitude by 0.05° longitude grid, and defined this as the spatial probability density of spawning location for that larva.

Instead of only backtracking larvae to estimate where they originated, we also sought to estimate the relative spawning output needed to yield the abundances and ages of larvae in our samples. To do this, it is necessary to scale the spatial probability density of spawning location by the number of eggs that would need to be spawned to lead to the observation of each larva, which depends on the age of the larva and the daily mortality rate. Instantaneous daily mortality rates (Z) for fish larvae strongly depend on temperature, and a meta-analysis across a wide range of latitudes, species, and ecosystems found that Z = 0.0256 + 0.0123 T, where T is the temperature that larvae experience^61^. We used the average temperature in the upper 50 m at each station to calculate the Z for larvae collected at that station: for our data, Z ranges from 0.357 to 0.399 day^−1^. This yielded a realistic estimate of the number of eggs that must have been spawned in order to lead to the observed catch.

For each larva, we scale the spatial probability density plot by the estimated number of eggs spawned, to generate the distribution of relative spawning output for that particular larva. We then sum the relative spawning output distributions across all larvae in a given year and taxon to produce maps that show the relative spawning output for the full collections of tuna larvae. Larval catch numbers and the resulting estimates of relative spawning output for each station are presented in Table S1.

## Supplementary information

Supplemental materials to Hernández, et. al: Evidence and patterns of tuna spawning inside a large no-take Marine Protected Area

## Data Availability

The datasets generated and analyzed during the current study are available from the corresponding author upon reasonable request. Genetic sequences were submitted to GenBank and can be found at accession numbers MK567307-MK567376.
