# Supplementary material for: Evidence and patterns of tuna spawning inside a large no-take Marine Protected Area
Source: Sci Rep. 2019 Jul 24;9:10772. doi: 10.1038/s41598-019-47161-0 (PMC6656763; doi:10.1038/s41598-019-47161-0)
Supplement: Supplementary file 1 — Supplemental materials to Hernández, et. al: Evidence and patterns of tuna spawning inside a large no-take Marine Protected Area [file 41598_2019_47161_MOESM1_ESM.docx]

**Supplemental materials to Hernández, et. al: Evidence and patterns of tuna spawning inside a large no-take Marine Protected Area**

**Christina M. Hernández**, Jan Witting, Ciara Willis, Simon Thorrold, Joel K. Llopiz, Randi D. Rotjan

*Manual inspection of DNA barcoding sequences for species identification of* Thunnus *larvae*

Of the 36 DNA barcodes recovered for *Thunnus* spp. larvae, 11 were too short for confident identification in the Barcode of Life Database (bold.org). For 4 of these, manual inspection of the sequence data enabled positive species identification through the use of diagnostic genetic markers and comparison with reference sequences^1^. The other 7 reads were very short (~230 bp) and did not include both of the two necessary markers to perform a definite species identification . Instead, these individuals were identified as members of the *Neothunnus* subgenus, which includes *T. albacares*, *T. tonggol*, and *T. atlanticus*^2^. Although these reads lacked a diagnostic genetic marker to differentiate among species within the *Neothunnus* subgenus, based on the distribution of *T. tonggol* in the Indian Ocean and *T. atlanticus* in the Atlantic Ocean, we have concluded that those larvae are *T. albacares*.

*Genetic barcoding insights for morphological identification*

After receiving DNA barcoding results for larvae from 2015 that were still intact (the eye had been sent for barcoding instead of tail tissue), the 16 larvae that were identified with barcoding were re-examined to see if morphological characters could be used to differentiate the two species. Bigeye tuna often have a distinct pair of pigment spots on the lower jaw and 1 or 2 pigment spots on the ventral edge about 7 or 8 myomeres anterior to the caudal fin base, while yellowfin tuna are expected to have a single pigment spot on the lower jaw and no ventral body pigment^3^. However, amongst the tuna larvae that were genetically identified as bigeye and yellowfin, these characters were not found to be reliable. Jaw pigments can be quite similar and are easily rendered indistinguishable by degradation, and many of the individuals identified as yellowfin tuna had small ventral or midline pigment spots. Larvae that were genetically identified as either species could have a single distinct pigment spot dorsal to the notochord tip.

*Length at age analyses*

Otoliths were extracted from individual larvae with dissecting pins; both sagittae and lapillae were extracted and placed flat side down on a glass microscope slide in Type B immersion oil. Otoliths were imaged with a Leica DM2500 compound microscope with an oil-immersion 100X objective lens; images were taken with a Leica MC120 HD Camera and the Leica Application Suite software. Images were calibrated using a stage micrometer. Otoliths were read in ImageJ using the ObjectJ plug-in. All extractions and reads were performed by the same reader. All otoliths for a given year were read once, the order of the images was shuffled, and they were read again. If the two reads yielded ages within +/- 1 day, the second read was retained. If the two reads differed by more than 1 day, a third read was performed. If the third read agreed to within +/- 1 day of either the first or second read, then the third read was retained. If the third read differed by more than 1 day from both the first and second reads, then that fish was not retained in age analyses. Otoliths were extracted and read from 118 tuna larvae total: 36 and 30 skipjack and 25 and 27 *Thunnus* spp. from 2015 and 2016, respectively. Otolith reads were retained for 32 and 30 skipjack larvae and 21 and 26 *Thunnus* spp. larvae from 2015 and 2016, respectively.

Linear least squares regression was used to construct a model of larval length at age for each taxon-year pair (e.g. skipjack tuna larvae collected in 2015) and for the pooled data for a taxon across years (e.g. all aged skipjack tuna larvae) (**Figure S1**). Because of the strong region-wide difference in temperature and productivity between 2015 and 2016, an ANOVA test was used to investigate the difference in the slopes of the year-wise linear regressions. This ANOVA test did not find a significant effect of year on larval length for skipjack tuna larvae (p > 0.2), and there was a marginally significant effect for *Thunnus* spp. larvae (p = 0.06). We found that average growth rates over the first two weeks of life are 0.45 and 0.37 mm per day for skipjack tuna larvae and *Thunnus* spp. larvae, respectively (**Figure S1**).


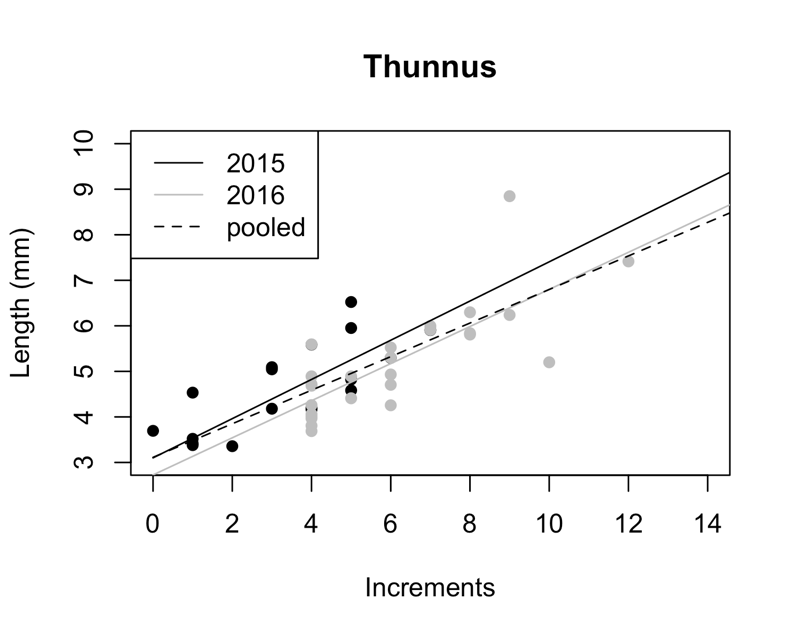

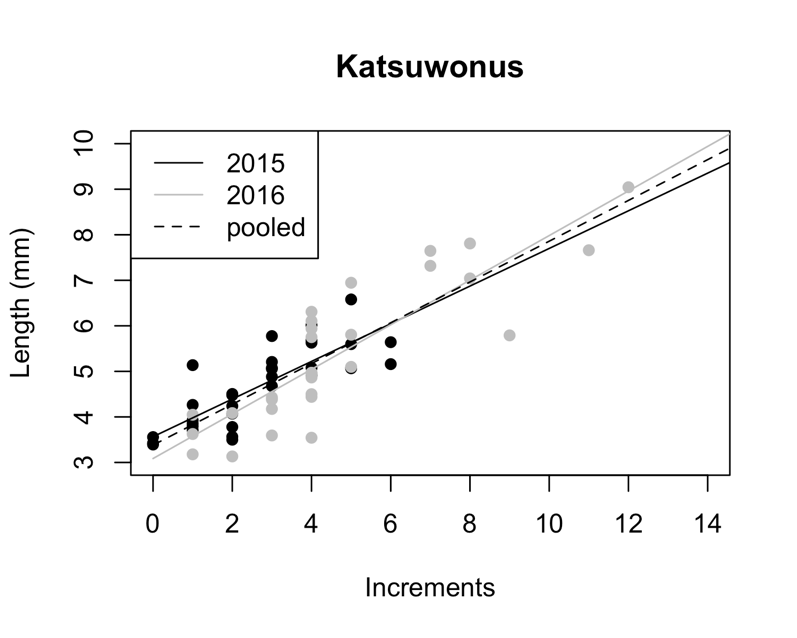


Figure S1: Larval growth curves for skipjack and *Thunnus* spp*.* larvae. Larval size-at-age for skipjack (A) and *Thunnus* (B) larvae in the first 12 daily increments (i.e., days of life following the onset of exogenous feeding). Data from individuals collected in 2015 is plotted with dark black circles and data from individuals collected in 2016 is plotted with grey circles. Linear regression lines for each year of data are plotted in the color to match the circles, and a dashed line shows the regression relationship when data for both years are pooled.

**a**

**b**

L = 3.38 + 0.45*DI

L = 3.11+ 0.37*DI

*HyCOM validation*

Before using a model for particle tracking, it is important to analyze how well that model represents the hydrodynamics of the study region. The Hybrid Coordinate Ocean Model (HyCOM) is a standard global ocean hydrodynamic model, and its data-assimilation procedures ensure quite good representation of the dynamics in any region with adequate observational coverage. However, tropical oceans can be difficult to represent in global models because some of the physical assumptions that govern these models are weaker at low latitudes.

Temperature and salinity are often used for model validation instead of current velocities, because the temporal and spatial scales of a model like HyCOM is difficult to compare with data from a shipboard acoustic Doppler current profiler (ADCP). However, temperature and salinity will generally track large-scale differences in water masses and, therefore, are signals that are useful for validating HyCOM. The model runs that are used for larval backtracking simulations are HyCOM GLBu0.08 (global velocity at 0.08-degree resolution) experiments 91.1 (2015) and 91.2 (2016 and 2017).

For this study, temperature and salinity from CTD casts are compared with the nearest-neighbor HyCOM point in longitude-latitude space. CTD data were processed aboard the *SSV Robert C. Seamans* using SeaBird Electronics software. The 2015 and 2016 data were provided at a vertical resolution of less than 1 m; the 2017 data were provided at a vertical resolution of 5 m. For comparison with HyCOM, CTD data are binned to the same depth levels as HyCOM (when possible): 0, 2, 4, 6, 8, 10, 12, 15, 20, 25, 30, 35, 40, 45, 50, 60, and 70 m depth. HyCOM depths extend to 5000 m, but the only dynamics pertinent to this study are in the upper ocean, where the larval tuna trajectories are simulated. Temperature and salinity measurements from the CTD are averaged, with the bin edges set at the midpoints between the HyCOM depth levels. For example, CTD data between 11 and 13.5 m is averaged to create an estimate of the temperature and salinity at 12 m depth. A number of stations (4 in 2015, 41 in 2016) had very low salinity values at the measurement nearest to the ocean surface (~0.6 m water depth). This could be real data, caused by a lens of freshwater (i.e., rain) at the surface, or it could be due to startup effects, since these instruments are generally rinsed with freshwater between deployments. Either way, it is unlikely for HyCOM to match them, so these surface points were removed from the comparison. In 2017, the CTD data were processed differently on board, and the depth bins available for HyCOM validation are coarser. The HyCOM model output has missing data on 2 dates for which CTD casts were performed in PIPA (July-15-2016 and August-4-2017).

The HyCOM validation was quantified using 3 statistical measures: the root-mean-square temperature difference between modeled and observed temperatures, $rmsd=\sqrt{\sum_{i=1}^{N} \frac{{(M_{i}-O_{i})}^{2}}{N}}$, the mean relative temperature difference (i.e., absolute difference divided by the observed value and averaged over all data points),$re=\frac{1}{N}\sum_{i=1}^{N} \left| M_{i}-O_{i} \right|/|O_{i}|$, and the cross correlation coefficient,$cc=\frac{\sum_{i=1}^{N} (M_{i}-\bar{M})(O_{i}-\bar{O})}{\sqrt{\sum_{i=1}^{N} {(M_{i}-\bar{M})}^{2}}\sqrt{\sum_{i=1}^{N} {(O_{i}-\bar{O})}^{2}}}$ . In the above notation, M represents a model-based value and O represents an observation value; N is the number of data points; overbar denotes average of all data points; and vertical bars denote absolute value. If the model and observations were all perfectly matching, then rmsd and re would both be equal to 0, and the cc would be equal to 1. As points move off of the 1:1 line, rmsd and re increase and cc decreases.

**Figure S2** shows that the relationship is strong for both temperature and salinity but both variables have a handful of outliers. For salinity, the *rmsd* is 0.3, the *re* is 0.006, and the *cc* is 0.715. For temperature, the *rmsd* is 0.465, the *re* is 0.01, and the *cc* is 0.798.

*CTD data:*


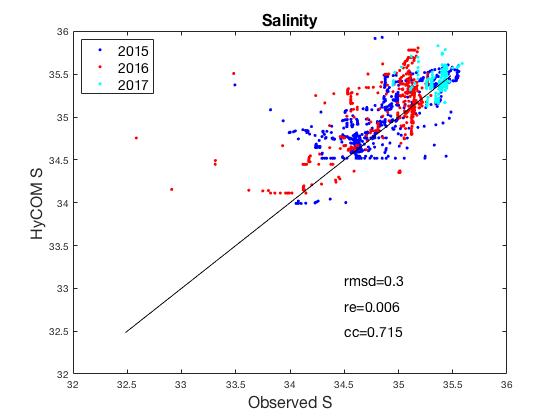

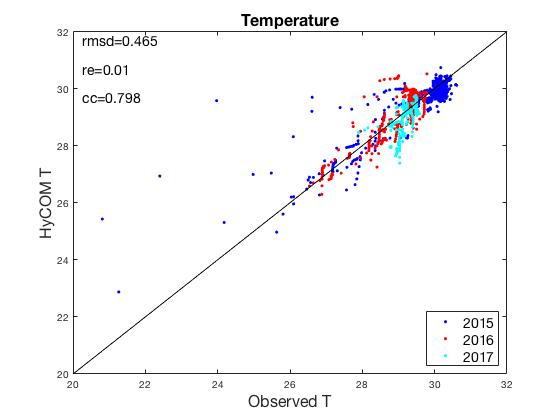


Figure S2: HYCOM validation plots. Observed values from CTD casts are plotted against HYCOM output values for salinity (a) and temperature (b). The different colors represent the years of sampling. RMSD, RE, and CC values are also shown.

**a**

**b**


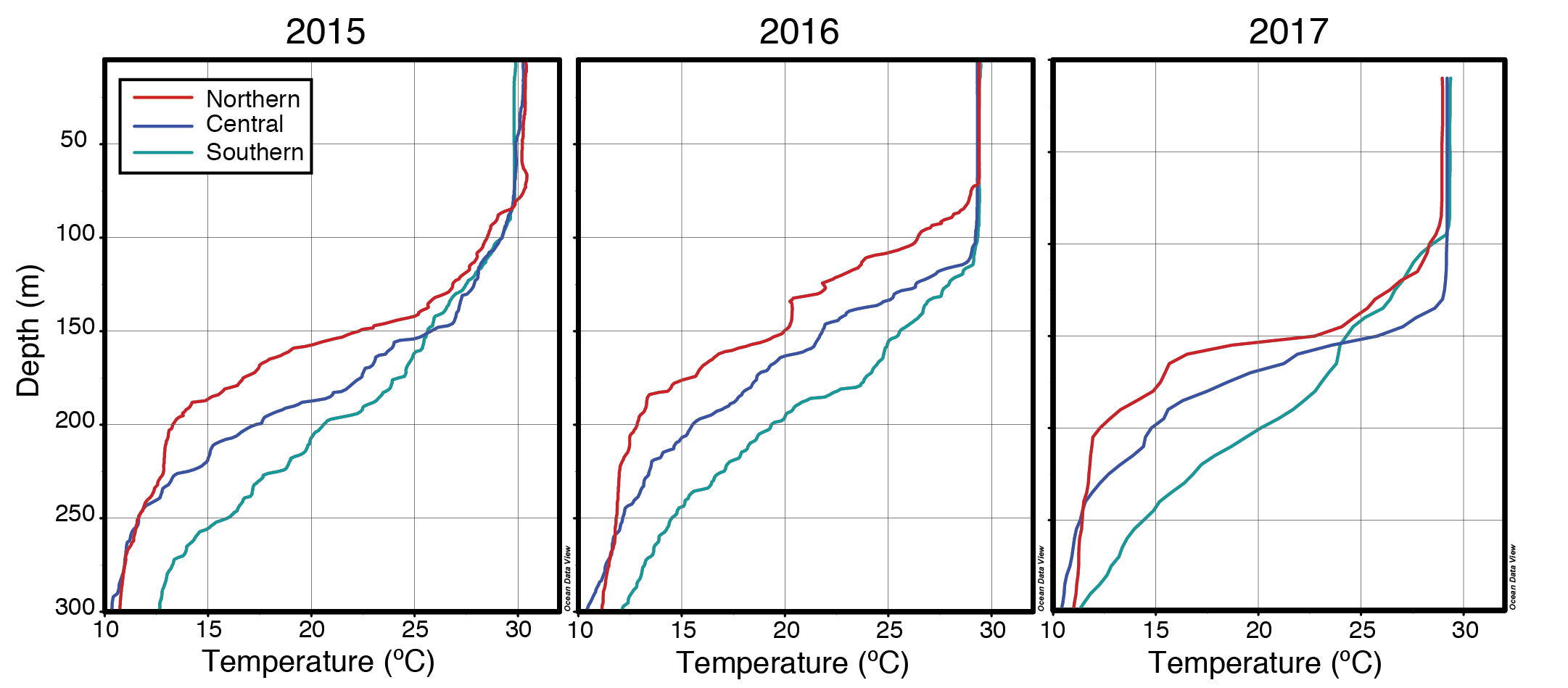
 Figure S3 shows temperature profiles in the upper 300m for stations in the western half of PIPA, in the portion of the transect that extended roughly north-south.

**a**

**b**

**c**

Figure S3: Temperature-at-depth profiles for (a) 2015, (b) 2016, and (c) 2017. Profiles are plotted for stations near the northern boundary, central latitude, and southern boundary of PIPA for each year.

Table S1: Information on stations with non-zero catch of larval tunas. Possible net tows include a deep Tucker Trawl to 100 m (TTD), a shallow Tucker Trawl to 50 m (TTS), and a neuston tow (NT). Stations that are outside of PIPA are indicated with a *. For each station, the average temperature (upper 50 m) from hydrocast data, latitude, and longitude are reported. For the two taxa (*Katsuwonus pelamis* and *Thunnus* spp.), the number of larvae collected, the abundance in number per 10 m^2^, and the spawning output needed to lead to the observed catch number. Additionally, the number of genetically identified larvae are reported for *Thunnus* spp. larvae in parentheses (*T. albacares* first, *T. obesus* second).

| **Year** | **Station** | **Nets Used** | **Mean T to 50m (C)** | **Station Latitude (ºN)** | **Station Longitude (ºE)** | ***Katsuwonus pelamis*** | | | ***Thunnus spp.*** | | |
| --- | --- | --- | --- | --- | --- | --- | --- | --- | --- | --- | --- |
|  |  |  |  |  |  | **N collected** | **N per 10 m^2^** | **Spawning output needed** | **N collected** | **N per 10 m^2^** | **Spawning output needed** |
| **2015** | 17* | NT | 30.00 | 1.87 | -166.61 | 1 | 0.006 | 2 | --- | --- | --- |
|  | 18* | TTD, NT | 30.02 | 0.67 | -167.28 | 101 | 42.50 | 1333 | 1 (1,-) | 0.42 | 2 |
|  | 20* | TTD, NT | 30.15 | -0.49 | -168.91 | 2 | 0.64 | 27 | 4 (3,-) | 1.29 | 101 |
|  | 21 | TTD, NT | 30.08 | -1.02 | -169.76 | --- | --- | --- | 9 (3,-) | 3.7 | 114 |
|  | 22 | TTD, NT | 30.21 | -1.51 | -170.08 | --- | --- | --- | 5 (4,-) | 2.81 | 55 |
|  | 23 | TTD, NT | 30.16 | -1.83 | -170.38 | 26 | 9.81 | 251 | 6 (3,-) | 2.13 | 76 |
|  | 24 | TTD, NT | 30.09 | -2.16 | -170.57 | 2 | 1.16 | 54 | --- | --- | --- |
|  | 25 | TTD, NT | 30.08 | -2.47 | -170.85 | 2 | 0.64 | 8 | 1 (-,1) | 0.64 | 11 |
|  | 26 | TTD, NT | 30.01 | -3.12 | -171.14 | 1 | 1.05 | 5 | 2 (1,-) | 2.1 | 9 |
|  | 28 | TTD, NT | --- | -3.01 | -171.39 | 1 | 0.38 | 35 | 1 | 0.38 | 3 |
|  | 29 | TTD, NT | 30.12 | -2.85 | -171.74 | 3 | 1.36 | 31 | 1 (1,1) | 0.46 | 16 |
|  | 31 | TTD, NT | 30.11 | -3.37 | -171.66 | 1 | 0.004 | 2 | --- | --- | --- |
|  | 35 | TTD, TTS, NT | 30.01 | -3.74 | -172.85 | 1 | 0.39 | 7 | 1 (1,-) | 0.72 | 11 |
|  | 42 | TTD, TTS, NT | 30.33 | -1.44 | -174.49 | 1 | 0.83 | 3 | 4 (1,2) | 1.4 | 56 |
|  | 44 | TTD, TTS, NT | 30.29 | -1.66 | -174.68 | --- | --- | --- | 2 (-,2) | 0.009 | 4 |
|  | 56 | TTD, TTS, NT | 30.24 | -2.93 | -174.86 | --- | --- | --- | 1 | 0.32 | 11 |
|  | 57 | TTD, TTS, NT | 30.22 | -3.41 | -174.66 | 1 | 0.74 | 3 | --- | --- | --- |
|  | 61 | TTD, TTS, NT | 29.98 | -4.71 | -174.55 | 2 | 0.72 | 23 | --- | --- | --- |
|  | 62 | TTD, TTS, NT | 29.95 | -5.14 | -174.24 | 8 | 1.94 | 56 | --- | --- | --- |
|  | 64 | TTD, TTS, NT | 29.83 | -6.47 | -173.61 | 10 | 1.38 | 77 | 1 | 0.36 | 3 |
| **2016** | 22* | TTD, TTS, NT | 28.12 | -0.49 | -170.15 | --- | --- | --- | 1 | 0.27 | 9 |
|  | 23 | TTD, TTS, NT | 28.20 | -1.05 | -170.34 | 1 | 0.7 | 184 | --- | --- | --- |
|  | 24 | TTD, TTS, NT | 28.57 | -1.47 | -170.40 | 2 | 0.53 | 20 | --- | --- | --- |
|  | 25 | TTD, TTS, NT | 28.95 | -2.06 | -170.44 | 52 | 11.59 | 632 | 2 | 1.52 | 52 |
|  | 26 | TTD, TTS, NT | 29.64 | -2.50 | -170.85 | 2 | 0.66 | 243 | 2 | 1.29 | 78 |
|  | 27 | TTD, TTS, NT | 29.32 | -2.93 | -170.51 | 180 | 40.75 | 1537 | 2 | 1.2 | 113 |
|  | 28 | TTD, TTS, NT | 29.18 | -3.11 | -171.14 | 2 | 1.14 | 79 | 2 | 0.05 | 25 |
|  | 29 | TTD, TTS, NT | 29.24 | -3.02 | -171.37 | 1 | 0.35 | 2 | --- | --- | --- |
|  | 31 | TTD, TTS, NT | 29.45 | -2.86 | -171.76 | 2 | 0.61 | 17 | 2 | 0.45 | 43 |
|  | 32 | TTD, TTS, NT | 29.30 | -3.51 | -171.73 | 17 | 2.32 | 87 | 4 | 0.78 | 40 |
|  | 33 | TTD, TTS, NT | 29.32 | -4.18 | -172.29 | 27 | 8.97 | 447 | 1 | 0.67 | --- |
|  | 34 | TTD, TTS, NT | 29.41 | -4.57 | -172.29 | --- | --- | --- | 1 | 0.5 | 3 |
|  | 37 | TTD, TTS, NT | 29.32 | -3.54 | -173.59 | 1 | 0.23 | 7 | 5 (-,2) | 0.71 | 105 |
|  | 38 | TTD, TTS, NT | 29.43 | -3.00 | -173.94 | 2 | 0.51 | 169 | 1 | 0.51 | 48 |
|  | 39 | TTD, TTS, NT | 29.52 | -2.55 | -174.34 | --- | --- | --- | 1 | 0.35 | 106 |
|  | 40 | TTD, TTS, NT | 29.47 | -1.97 | -174.66 | --- | --- | --- | 3 (-,2) | 0.38 | 205 |
|  | 41 | TTD, TTS, NT | 29.37 | -1.59 | -174.82 | --- | --- | --- | 1 (-,1) | 0.68 | 71 |
|  | 42 | TTD, TTS, NT | 29.42 | -1.83 | -175.05 | --- | --- | --- | 2 (-,1) | 0.5 | 275 |
|  | 45 | TTD, TTS, NT | 29.33 | -3.61 | -174.74 | --- | --- | --- | 1 (-,1) | 0.25 | 10 |
|  | 47 | TTD, TTS, NT | 29.35 | -4.73 | -174.62 | 1 | 0.3 | 2 | --- | --- | --- |
|  | 48 | TTD, TTS, NT | 29.39 | -5.86 | -174.58 | --- | --- | --- | 1 (-,1) | 0.27 | 15 |
|  | 49 | TTD, TTS, NT | --- | -6.15 | -174.30 | 1 | 0.38 | 15 | 1 | 0.61 | 10 |
|  | 50 | TTD, TTS, NT | 29.45 | -6.42 | -173.70 | --- | --- | --- | 2 (-,1) | 0.39 | 32 |
| **2017** | 9* | TTD, NT | 29.50 | -6.29 | -169.32 | 8 | 7.12 | 25 | 1 (1,-) | 0.89 | 3 |
|  | 11* | TTS, NT | 29.14 | -4.76 | -169.19 | 2 | 1.39 | 44 | 1 | 0.69 | 7 |
|  | 13 | TTS, NT | 29.11 | -3.79 | -169.81 | 2 | 0.98 | 53 | 2 (-,1) | 0.62 | 17 |
|  | 14 | TTD, TTS, NT | 28.96 | -3.22 | -170.22 | --- | --- | --- | 1 | 0.46 | 67 |
|  | 15 | TTD, TTS, NT | 29.11 | -3.13 | -171.16 | 3 | 3.14 | 32 | --- | --- | --- |
|  | 16 | TTD, TTS, NT | 29.03 | -3.02 | -171.40 | 6 | 3.73 | 100 | --- | --- | --- |
|  | 17 | TTD, TTS, NT | 28.83 | -2.84 | -171.75 | 1 | 1.46 | 2 | --- | --- | --- |
|  | 19 | TTS, NT | 29.11 | -4.10 | -171.98 | 1 | 0.24 | 32 | --- | --- | --- |
|  | 20 | TTD, TTS, NT | 29.15 | -4.28 | -172.01 | 1 | 0.004 | 2 | --- | --- | --- |
|  | 22 | TTS, NT | 29.24 | -4.42 | -172.35 | 4 | 0.85 | 146 | --- | --- | --- |
|  | 23 | TTD, TTS, NT | 29.24 | -4.04 | -173.03 | 3 | 0.65 | 25 | --- | --- | --- |
|  | 24 | TTD, TTS, NT | 29.24 | -3.47 | -173.61 | 1 | 0.19 | 2 | --- | --- | --- |
|  | 26 | TTD, TTS, NT | 29.01 | -2.17 | -174.40 | 5 | 0.89 | 205 | --- | --- | --- |
|  | 27 | TTD, TTS, NT | 28.96 | -1.81 | -174.67 | 2 | 0.62 | 28 | --- | --- | --- |
|  | 28 | TTD, TTS, NT | 29.07 | -2.44 | -174.80 | 1 | 0.34 | 2 | 1 | 0.34 | 21 |
|  | 29 | TTD, TTS, NT | 29.18 | -2.99 | -174.93 | 20 | 4.74 | 170 | --- | --- | --- |
|  | 30 | TTD, TTS, NT | 29.20 | -3.80 | -174.65 | 8 | 1.23 | 64 | 1 (1,-) | 0.2 | 22 |
|  | 31 | TTD, TTS, NT | 29.44 | -4.67 | -174.43 | 2 | 0.35 | 22 | --- | --- | --- |
|  | 32 | TTD, TTS, NT | 29.40 | -4.67 | -174.58 | 7 | 1.49 | 31 | 1 | 0.4 | 5 |
|  | 33 | TTD, TTS, NT | 29.48 | -5.23 | -174.26 | 2 | 0.53 | 9 | --- | --- | --- |
|  | 34 | TTD, TTS, NT | 29.45 | -5.12 | -174.18 | 3 | 1.47 | 29 | --- | --- | --- |

Supplemental Information Citations:

1. Richardson, D. E., Vanwye, J. D., Exum, A. M., Cowen, R. K. & Crawford, D. L. High-throughput species identification: from DNA isolation to bioinformatics. *Mol. Ecol. Notes* **7,** 199–207 (2007).

2. Chow, S., Nakagawa, T., Suzuki, N., Takeyama, H. & Matsunaga, T. Phylogenetic relationships among *Thunnus* species inferred from rDNA ITS1 sequence. *J. Fish Biol.* **68,** 24–35 (2006).

3. Nishikawa, Y. & Rimmer, D. W. Identification of larval tunas, billfishes and other scombroid fishes (Suborder Scombroidei): an illustrated guide. *CSIRO Reports* **186,** 1-20 (1987).
